# Supplementary material for: COVID-19 Vaccination in Young People with Functional Neurological Disorder: A Case-Control Study
Source: Vaccines (Basel). 2022 Nov 28;10(12):2031. doi: 10.3390/vaccines10122031 (PMC9782633; doi:10.3390/vaccines10122031)
Supplement: Supplementary file 1 [file vaccines-10-02031-s001.zip › vaccines-1989844-supplementary.pdf]

**Table S1.** Adjunctive medication for comorbid disorders or symptoms in cases 1–6 used during their treatment for FND.

| | Medication | Indication |
|---|---|---|
| Case 1 | Escitalopram | Comorbid anxiety |
| | Melatonin at night | For help with sleep initiation |
| | Quetiapine at night | For help with sleep initiation and down-regulation of arousal |
| | Movicol | Maintenance bowel function |
| | Sodium Chloride | Comorbid POTS |
| | Vitamin D | Low vitamin D |
| | Iron Tablets | Low iron |
| Case 2 | Escitalopram | Comorbid anxiety |
| | Clonidine | For down-regulation of arousal during the day and help with sleep initiation at bedtime |
| Case 3 | Fluoxetine | Major depression |
| | Quetiapine at night | For help with sleep initiation (until fluoxetine took effect) |
| Case 4 | Escitalopram | Comorbid anxiety |
| | Propranolol | Comorbid POTS |
| | Clonidine at night | For help with sleep initiation and down-regulation of arousal |
| Case 5 | Sertraline | Comorbid depression |
| | Guanfacine | Downregulation of arousal |
| | Quetiapine | Downregulation of arousal during day, sleep initiation night |
| | Omeprazole | Comorbid functional gut disorder (nausea) |
| | Movicol | Comorbid functional gut disorder (constipation) |
| Case 6 | Dexamphetamine | Narcolepsy (type 2) |
| | Fluvoxamine | Comorbid anxiety and depression |
| | Melatonin | To help with sleep initiation at night |
| | Olanzapine | To help contain arousal during the day and as needed doses to manage episodes of extreme distress or prolonged functional seizures |
| | Ondansetron | Comorbid functional gut disorder (nausea) |

**Supplementary Text Box S1.** COVID-19 vaccine rollout in Australia.

The Australian government's national COVID-19 vaccine rollout strategy was commenced, for some adults, in late February 2021. In early August 2021, the Australian Technical Advisory Group on Immunisation (ATAGI) recommended the Comirnaty (Pfizer) vaccine for children 12–15 years old with specified medical conditions that increase their risk of severe COVID-19, for Aboriginal and Torres Strait Islander children, and for children in remote communities. Those with severe mental health conditions were included in this population [13].

In late August 2021, the recommendation for vaccination, with either Comirnaty (Pfizer) or Spikevax (Moderna),was extended to include all children from 12 years. This recommendation followed the phase III trial data that reported Pfizer vaccine efficacy against COVID-19 from 7 days after the second dose of 100% (95% CI, 78.1–100) [14]. Trial data for Moderna in the paediatric populations reported vaccine efficacy against symptomatic COVID-19 from 14 days after the second dose of 93.3% (95% CI, 47.9–99.9) [15]. Both vaccine formulations were also shown to be immunogenic.

In early December 2021, the ATAGI recommended vaccination with the paediatric Pfizer COVID-19 vaccine for all children aged 5–11 years. In this staged rollout the indirect benefits of vaccinating the paediatric population were also strongly considered—including the reduced likelihood of school closures and disrupted extracurricular and social activities.

**Supplementary Text Box S2.** Functional somatic symptoms caused by hyperventilation and the associated low PCO2 and respiratory alkalosis

| Location of the neurophysiological changes/Symptoms experienced | Mechanism |
|---|---|
| **In the body proper** | |
| **Arms, legs, and face**<br>Pins and needles in the hands and feet<br>Twitchy muscles, or even spasms in the hands or feet (carpopedal spasms) | Activation of sensory and skeletomotor nerves |
| **Chest**<br>Hyperventilation-induced chest pain. | Vasoconstriction of arteries supplying blood and oxygen to the heart results in decreased blood flow and delivery of oxygen |
| **Whole body (energy regulation in the body as a whole)**<br>Fatigue | Sustained increases in respiratory rate over time—which increase the body's demand for energy—contribute to symptoms of fatigue |
| **In the brain** | |
| **Phase 1 of the biphasic hyperventilation response**<br>Excitation of the cortex<br>Difficulty in thinking clearly (impaired prefrontal cortex function)<br>Children and adolescents prone to functional seizures—temporary dysregulation of neural networks—are at increased risk of triggering a functional seizure [34] | Hyperventilation initially causes cortical excitation (phase 1); that is, it increases brain arousal and neuron excitability<br>The prefrontal cortex is particularly sensitive to cortical arousal [35] |
| **Phase 2 of the biphasic hyperventilation response**<br>Some degree of cerebral hypoxia<br>Symptoms of cerebral hypoxia include the following:<br>• dizziness<br>• changes in vision (ranging from changes in colour to complete blackout)<br>• decreasing levels of consciousness (to loss of consciousness)<br>• loss of muscle tone (potentially culminating in falling) | If hyperventilation continues beyond the short term (phase 2), it causes (to an extent that varies from individual to individual) vasoconstriction of arteries supplying blood and oxygen to the brain, resulting in decreased blood flow and delivery of oxygen, and in some degree of cerebral hypoxia<br>The prefrontal cortex is particularly sensitive to hypoxia |

**Supplementary Text Box S3.** Common positive (rule-in) neurological signs found on physical examination in children with functional motor and sensory symptoms*

| Neurological symptom | Neurological signs that the neurologist may use to support a diagnosis of FND |
|---|---|
| Across symptoms | • Symptoms are more marked when the child attends to them and less marked when the child's attention is directed elsewhere<br>• Symptoms are variable across contexts (e.g., a child presenting with visual loss can use her mobile phone but cannot see the text that she needs to read in the classroom; a child's gait difficulty is present when she walks forward but not when she turns around; tremor in a limb is less when the child is distracted by the neurologist) |
| Gait difficulty | • A swaying gait or apparent loss of balance with a narrow-base gait<br>• Each foot is lifted off the ground as if requiring great effort and put back down as if requiring great effort<br>• The child walks with bent knees (which requires more strength than normal walking) |
| Weakness (generalized or partial) | • Discordance between strength or functional ability of the child's affected body part on formal examination and during routine tasks (e.g., moving around on the hospital bed)<br>• Limb weakness not conforming to an anatomical distribution (e.g., arm and leg weakness on opposite sides of the body) |
| Tremor | • Variable distribution or frequency of the child's tremor when examined at different times<br>• The child's tremor changes with contralateral body movements (entrainment) |
| Sensory symptoms (pain excluded) | • Sensory symptoms not conforming to a dermatomal distribution<br>• Hemisensory loss with a sharp midline distribution |
| Visual loss | • Tunnel vision<br>• Preserved response to a "menace reflex" (the rapid approach of an object) |

© Shekeeb Mohammad and Kasia Kozlowska 2022.

* For a more comprehensive list of rule-in signs, see Kozlowska and Mohammad 2022 [38].

**Supplementary Text Box S4.** Summary of the measures used in the study

| Measure | Description |
|---|---|
| RAHC-GAF | The Royal Alexandra Hospital for Children Global Assessment of Function is the DSM-IV-TR GAF modified to include functional impairment secondary to physical illness. The scale has 100 points and 10 categories (10 points each). Healthy controls fall into the upper two brackets "superior in all areas" (score 91–100) or "good in all areas" (score 81–90). Lower values (and brackets) mark functional impairment of increasing severity. Patients with physical or psychological impairment fall into the lower brackets (score <81). |
| DASS-21 | The Depression Anxiety and Stress Scales is a validated measure of perceived distress in paediatric populations [39,40]. |
| ELSQ | The Early Life Stress Questionnaire is a checklist of 19 stress items—and an option for elaboration—based on the Child Abuse and Trauma Scale [41]. Twelve items pertain to relational stressors, including the following: bullying; physical abuse; sexual abuse; emotional abuse; neglect; parental separation; loss by separation; loss by death; family conflict; severe illness of a family member; domestic violence; and other. Other items pertain to birth complications, life-threatening/severe illness, war trauma, and natural disasters. Participants record if they have or have not experienced the given stressor and the age period during which the stressor has been experienced. |

**Supplementary Text Box S5.** Script used to collect follow-up information from patients discharged from the mind-body program and from controls

Akin to our clinical assessments and previous follow-ups, wherever possible, the phone interview was done with the young person and a parent. Below is the script that was used to guide the interview.

*I am NL, the current registrar on the mind-body team. I am ringing everyone in the FND research study to collect follow-up data. Is it Ok if I ask a few questions?*

**Open-ended question 1—asked as part of standard follow-up for FND research program**

For patient: *How is [patient name] travelling since the last follow-up phone call?*

For control: *How is [control name] travelling? Have there been any changes in {control's name] physical or mental health since he/she participated in the study?*

Prompts for patients if not previously covered:

Are [patient name's] FND symptoms ongoing or resolved?
If ongoing: What are your current symptoms and how frequent are they?
Does [patient name] have any ongoing functional somatic symptoms (other than FND)?
Does [patient name] have any ongoing mental health concerns?
Is [patient's name] attending school, university, or work?

For patients and controls: *We are asking everyone in the study about their experience with the COVID-19 vaccine. Have you been vaccinated? How many doses did you have (and which vaccine)?*

**Open-ended question 2 (COVID vaccine–related)**
For patients and controls: *Did you have any issues with the vaccine?*

Prompts for patients and controls if the above question was answered in the positive:

Which vaccine formulation (Pfizer or Moderna) did you have?
What was the timing of symptom exacerbation/emergence in relation to the vaccine?
Was the issue after the first or second vaccination?
If the young person reported exacerbation of FND symptoms post vaccination:

- What was the frequency, severity and progression to date of symptoms?
- Consent requested for vignette and consent form distributed?

If the young person reported new FND symptoms post vaccination:

- Was the FND diagnosis confirmed by a medical practitioner (e.g., family doctor)?
- What was the frequency severity and progression to date of symptoms?
- Consent requested for vignette and consent form distributed?

For patients and controls: *And have you been sick with COVID?*
Did you get COVID before or after vaccination (or between vaccines)?

**Open-ended question 3 (COVID-related)**

For patients and controls: *What was your COVID illness like?*

For patients and controls: *Is there anything else you would like to tell me? Is there anything else I should have asked you about?*

**Supplementary Text Box S6.** Vaccination as a trigger for narcolepsy

Narcolepsy is a sleep disorder characterised by excessive daytime sleepiness [43,44]. With an onset that is most common in childhood [45], it affects approximately 20–50/100,000 children, with a slight male predominance [43,46]. Children with narcolepsy may also experience cataplexy, sleep paralysis, hypnogogic or hypnopompic hallucinations, and disturbed sleep during the night [43,45].

Currently, children with narcolepsy can be divided into two types based on the presence or absence of cataplexy [43,47]. Children with narcolepsy type 1 present with excessive daytime sleepiness, cataplexy, or low cerebral hypocretin levels. "One current hypothesis is that narcolepsy is related to the destruction of the specific brain area responsible for sleep and wake function and the loss of hypocretin, a neuropeptide in the brain" (p3) [43]. In particular, narcolepsy type 1 appears to be caused by the loss of hypocretin-1 (orexin) neurons in the lateral hypothalamus, reflected in low hypocretin levels (p4) [43] By contrast, children with narcolepsy type 2 do not have cataplexy, and their hypocretin levels are normal (or have not been formally tested). The aetiology of narcolepsy type 2 remains unknown. A subset of children with narcolepsy type 2 develop cataplexy later in the course of the illness.

The pathophysiology of narcolepsy type 1 is thought to involve an immune-mediated process in genetically predisposed individuals, resulting in orexin (hypocretin) deficiency following destruction of orexin-producing neurons in the hypothalamus. There is a strong association with the HLA-DQB1*06:02 genotype. Recognized environmental factors include infection (strep pyogenes, influenza, H1) and the H1N1 (Pandemrix, GSK) vaccine. It is hypothesised that a targeted immune-mediated (autoimmune) attack causes the specific degeneration of hypocretin neurons through the discovery of genetic associations (the HLA-DQB1*06:02 allele and the T-cell receptor $\alpha$ locus) [48]. Evidence for an immune-related aetiology has increased following epidemiological observations that H1N1 infection and vaccination are potential triggering factors [49–51]. While tiredness and sleepiness are commonly reported post-vaccination side effects, the first case report of narcolepsy triggered by the Pfizer/BioNTech vaccination against SARS-CoV-2 was in 2022 in the absence of HLA-associated alleles [52].

**Text Box 6.** Summary of key neuroimmune mechanisms

The following important neuroimmune mechanisms have been identified in the basic science literature (including animal models):

- There are "intricate functional and structural interactions between endothelial cells, glia, and neurons" (p 479) allowing for both long-range to short-range mechanisms of communication between the peripheral immune-inflammatory system and brain, and within the brain itself (p 498) [61].
- "In general, stress challenges the body, causing it to react by increasing activation of the sympathetic nervous system and HPA system. As part of the sympathetic nervous system response, norepinephrine[-mediated processes] . . . influence the immune system. The HPA system releases glucocorticoids that stimulate both anti- and pro-inflammatory responses. Acute stressors increase the release of glucocorticoids to reduce inflammation. Over time, chronic stress shifts the response to pro-inflammatory, allowing the organism to respond to sustained threat. Glucocorticoid levels or glucocorticoid responses in humans and animals are suppressed in some, but not all, individuals following chronic stress" (pp 26–28)[79].
- The mounting of an antibody response is associated with increased activity of the sympathetic nervous system and the HPA axis [61]: multiple components of the stress system are activated.
- Immunological memory appears to play an important role in the individual's response to subsequent stress (physical or psychological) [61,76,77]. Immunological memory is enhanced by glucocorticoid-related priming. Past glucocorticoid receptor activation on microglia has a priming effect that increases pro-inflammatory neuroimmune signals [76,80]. The result is that subsequent stress can trigger an amplified neuroinflammatory response in the brain. This amplified response can be expressed in physiological and behavioural sequelae (e.g., sickness behaviour) and potentially in neuropsychiatric sequelae.
- In animal models, acute or chronic exposure to stress before an inflammatory stimulus appears to prime the inflammatory response both in peripheral macrophages and in microglia—the resident macrophage population of the central nervous system [61]. It is possible that a history of chronic exposure to stress—cumulative stress—may likewise prime the stress system response to an inflammatory stimulus in humans.

**Supplementary Text Box 7.** Factors that contribute or are hypothesised to contribute to the neurobiological platform that maintains FND symptoms after they have been triggered by vaccination

| Psychological factors (changes in information processing) | Neurophysiological factors (changes in biological setpoints) |
|---|---|
| **Attention to symptoms**<br>Attention to functional symptoms amplifies them. The narrowing of attention to threat-related and emotion stimuli—and in particular body processes and symptoms—is a key feature of FND [6,7,94,95]. Attention to symptoms is hypothesised to function both as a top-down stressor that functions to maintain stress-system activation and as a factor that contributes to an aberrant predictive-coding process whereby the brain's predictive coding functions are disrupted (see right right-hand column). In the latter scenario, the real-time construction of motor action, sensory experience, and energy regulation is not well tailored to the immediate environment [9,10,96].<br>**Threat-related cognitions**<br>Threat-related cognitions—negative expectations, rumination, catastrophizing, and so on [7,33,42,93]—are common in young people with FND (even in the absence of comorbid anxiety or depression. They function as a top-down stressor that maintains stress-system activation and the neurobiological platform that supports FND symptoms.<br>**Ongoing anxiety, depression, or posttraumatic stress disorder** | **Ongoing activation/dysregulation of stress-system components**<br>In young people with FND, stress-system components are activated or dysregulated [3,69-72]. They are all thought to play a role in modulating aberrant connectivity seen in young people with FND.<br>**Ongoing activation of neural networks**<br>In young people with FND, neural networks are in a state of persistent activation [73-75]. This activation supports a shift from higher-order, cognitive processing to lower-order, emotion- and motor-reflexive modes of processing [34].<br>**Aberrant predictive coding**<br>The model of the brain as an active predictor [8-10] suggests that FND involves changes in implicit predictions pertaining to motor planning, sensory experience, and energy metabolism [9,10,96].<br>**Epigenetic reprograming of cells within neuron–glial networks**<br>Adult studies suggest changes in gene expression across multiple brain networks [11].<br>Stephenson and Baguley (2018) hypothesise that activation of primed (epigenetically reprogramed) glial cells—in the context of past stress—causes deactivation of synapses or reduced synaptogenesis, thereby maintaining motor and sensory symptoms [88]. Glial cell priming is also implicated in other stress-related disorders. |
